# Carbon Amendments Induce Shifts in Nutrient Use, Inhibitory, and Resistance Phenotypes Among Soilborne *Streptomyces*

**DOI:** 10.3389/fmicb.2019.00498

**Published:** 2019-03-27

**Authors:** José Pablo Dundore-Arias, Laura Felice, Ruth Dill-Macky, Linda L. Kinkel

**Affiliations:** Department of Plant Pathology, University of Minnesota, St. Paul, MN, United States

**Keywords:** *Streptomyces*, carbon amendments, soil mesocosms, resource use, antibiotic inhibition, antibiotic resistance

## Abstract

Carbon amendments are used in agriculture for increasing microbial activity and biomass in the soil. Changes in microbial community composition and function in response to carbon additions to soil have been associated with biological suppression of soilborne diseases. However, the specific selective impacts of carbon amendments on microbial antagonistic populations are not well understood. We investigated the effects of soil carbon amendments on nutrient use profiles, and antibiotic inhibitory and resistance phenotypes of *Streptomyces* populations from agricultural soils. Soil mesocosms were amended at intervals over 9 months with low or high dose solutions of glucose, fructose, a complex amendment, or water only (non-amendment control). Over 130 *Streptomyces* isolates were collected from amended and non-amended mesocosm soils, and nutrient utilization profiles on 95 different carbon substrates were determined. A subset of isolates (*n* = 40) was characterized for their ability to inhibit or resist one another. Carbon amendments resulted in *Streptomyces* populations with greater niche widths, and increased growth efficiencies as compared with *Streptomyces* in non-amended soils. Shifts in microbial nutrient use and growth capacities coincided with positive selection for *Streptomyces* antibiotic inhibitory phenotypes in carbon-amended soils, resulting in populations dominated by phenotypes that combine both antagonistic capacities and a generalist lifestyle. Carbon inputs resulted in populations that on average were more resistant to one another than populations in non-amended soils. Shifts in metabolic capacities and antagonistic activity indicate that carbon additions to soil may selectively enrich *Streptomyces* antagonistic phenotypes, that are rare under non-nutrient selection, but can inhibit more intensively nutrient competitors, and resist phenotypes with similar functional traits. These results shed light on the potential for using carbon amendments to strategically mediate soil microbial community assembly, and contribute to the establishment of pathogen-suppressive soils in agricultural systems.

## Introduction

Soil organic nutrient amendments are recognized for their potential to improve soil physicochemical properties and biological characteristics in agroecosystems, including carbon sequestration and nutrient availability, as well as microbial activity and biomass in the soil (Schutter and Dick, 2001; Gomez et al., 2006; Schlatter et al., 2009; Shi et al., 2011; Intanon et al., 2015; Regni et al., 2016). Similarly, organic amendments are known for their capacity to alter the composition and function of communities of soil microorganisms, and their use has also been linked to biological suppression of pathogens and enhanced plant performance (Larkin, 2015; Liu et al., 2015; Jaiswal et al., 2017). However, there is little predictive understanding of how manipulation of resource availability in the soil can contribute to selective enrichment of microbial populations that are beneficial for sustainable agricultural production. Here, we explore whether changes in resource availability via carbon substrates, constituents of plant root exudates and commonly used organic amendments, impose selection that would alter the phenotypic characteristics and species interactions of soil *Streptomyces*.

*Streptomyces* are Gram positive, filamentous bacteria characterized for their ubiquitous and abundant nature in the soil (Seipke et al., 2012). In agricultural settings, *Streptomyces* are known for their antagonistic capacities and ability to suppress diverse plant pathogens (Samac and Kinkel, 2001; Xiao et al., 2002; Otto-Hanson et al., 2013; Law et al., 2017). Although *Streptomyces* are widely recognized as potential biocontrol agents and prolific producers of a broad range of antibiotics, the production of secondary metabolites can be highly influenced by resource availability (D’Costa et al., 2006; Sánchez et al., 2010). Moreover, previous work suggests that competition for nutrients among coexisting *Streptomyces* mediates selection for antibiotic inhibitory capacity of soil *Streptomyces* (Kinkel et al., 2014). *Streptomyces* vary significantly in their ability to metabolize particular nutrients (Schlatter et al., 2013) and exhibit high specificity in antibiotic resistant and inhibitory interactions (Davelos et al., 2004), suggesting the potential for diverse resource and antagonistic competitive interactions among soil *Streptomyces*.

Green manure amendments have been shown to enhance the inhibitory activities of soil *Streptomyces*, but effects can be inconsistent (Wiggins and Kinkel, 2005a,b). Simple carbon amendments can also influence the densities and competitive phenotypes of *Streptomyces* from prairie soils (Schlatter et al., 2009). More recent work suggests that both plant host and community richness also significantly influenced *Streptomyces* inhibitory phenotypes in the soil (Bakker et al., 2010; Essarioui et al., 2016, 2017). The selective effects of plant diversity on soil *Streptomyces* populations were hypothesized to reflect variations in the amount and composition of plant-derived carbon compounds released into the soil. However, the specific selective impacts of carbon amendments on microbial species interactions and metabolic capacities remain poorly understood.

In this study, we amended agricultural field soils with labile carbon substrates over 9 months, simulating plant rhizodeposition over a crop-growing season. The goal of this work was to examine the effects of soil carbon amendments on nutrient use profiles, and antibiotic inhibitory and resistance phenotypes of *Streptomyces* populations from agricultural soils with a long history of conventional use. We hypothesized that sustained additions of carbon compounds to soil could impose selection that would shift the metabolic abilities and antagonistic interactions of soil *Streptomyces.* Understanding the role that soil carbon amendments play in mediating microbial nutrient competition and antagonistic interactions will provide valuable insight into mechanisms to selectively stimulate or repress indigenous soil microbial populations that impact the productivity and sustainability of agroecosystems.

## Materials and Methods

### Soil Collection and Processing

Soil was collected in fall 2012 from a field previously planted with potatoes at the University of Minnesota Sand Plain Research Farm in Becker, Minnesota. The loamy sandy soil has an average organic matter of 1.5% and nitrate of 15.2 ppm. Soil was collected from the freshly tilled field up to a depth of approximately 30 cm with a clean shovel to obtain a total volume of 45 L. Soil was stored at 4°C for 10 days before processing.

### Mesocosm Establishment

Soil was sieved through 0.64 cm mesh to remove residual plant material and rocks, and homogenized using a clean cement mixer. Four subsamples of soil were used for gravimetric measurement of soil moisture. Soil moisture cans were dried for 24 h in a convection oven at 105°C and subsequently cooled in a desiccator. Soil weight was measured before and after drying and used to calculate moisture content. Mesocosms were established in sterile 1 pint (0.47 L) canning jars each filled with 500 g dry weight of homogenized soil. Soil moisture levels in each jar were subsequently adjusted to 3.0 bars. Each jar was covered with two layers of sterile muslin cloth held on by a screw-cap metal ring to reduce the risk of microbial contaminants, but allowing free gas exchange. Mesocosms were arranged in a randomized complete block design, and incubated in a dark, dehumidified space at approximately 25°C.

Soil in each jar was amended for 9 months with a low or high dose sterile solution of glucose (Sigma-Aldrich Co.); fructose (Thermo Fisher Scientific Co.); a complex amendment consisting of a combination of glucose, fructose, and malic acid (Acros Organics); or with only sterile water (non-amended control). There were 4 replicated mesocosms for each treatment, resulting in a total of 28 mesocosms including the non-amended controls.

The carbon compounds used in this study were selected because they represent substances reported to be dominant constituents of plant litter and root exudates (Schutter and Dick, 2001; Dakora and Phillips, 2002; Orwin et al., 2006; Bradford et al., 2008). The low dose (equivalent to 250 g C/m^2^/year) amendments were chosen to approximate the annual carbon inputs to a highly productive prairie, and the high dose (equivalent to 750 g C/m^2^/year) to exceed this amount (Hernández and Hobbie, 2010). For the complex carbon amendment, an adjusted amount of each of the three nutrients was added so that each carbon source contributed an equal fraction of the total added carbon in that treatment. Amendments were made once every 14 days for the first 3 months, and once per month thereafter, with equal amounts of carbon added to the soil each time. Each time an amendment was added, soil moisture of the mesocosm was adjusted to maintain consistency over time. Briefly, ten jars were weighed individually using a Mettler PM 2000 balance (Mettle-Toledo). The differences from the original (moisture adjusted to 3.0 bars) weight and the current weight of each of the mesocosms were determined. Then the differences in weights were averaged across all mesocosms, and used to calculate the volume of water to be added to each jar in order to maintain the mesocosms at consistent moisture content. Non-amended control jars received the same volume of water used to add the carbon compounds into the soil, ranging from 23–37 ml, at each time point.

After the 9-month incubation, soil samples were collected from each mesocosm and used for soil total carbon (TC) analysis. Soil samples were analyzed by dry combustion at the Ecosystem Analysis Lab at University of Nebraska – Lincoln, using a Costech Analytical ECS 4010 (Costech Analytical Technologies Inc., Valencia, CA, United States).

### *Streptomyces* Isolates

After the 9-month incubation period, *Streptomyces* isolates were randomly collected from each treatment. Briefly, 5 g soil samples were collected from each jar, and dried overnight at room temperature (25°C) in a fume hood beneath 3 layers of sterile cheesecloth. Dried soil was then added to 50 mL of sterile water and shaken at 175 rpm for 60 min at 4°C. Soil suspensions were subsequently serially diluted, and the corresponding dilutions were plated on starch-casein agar (SCA) plates and incubated at 28°C for 5–7 days. Colonies exhibiting characteristic *Streptomyces* morphology were randomly selected from each plate using a numbered grid, sub-cultured on SCA plates, and incubated for 7 days at 28°C. Subsequently, a single pure colony was selected for each isolate and grown on oatmeal agar (OA) plates for 10–12 days. Spores of each isolate were collected into 2 mL of sterile 20% glycerol solution for storage, and maintained at -20°C. A total of 133 *Streptomyces* isolates were collected across all treatments, and an average of 20 (range 17–22) isolates were obtained from every treatment, with the isolates originating from at least 3 replicates (mesocosms) per treatment. These isolates were used for subsequent phenotypic characterization.

### Nutrient Use Characterization

Utilization of 95 distinct carbon sources by every individual *Streptomyces* isolate (*n* = 133) was determined using Biolog SF-P2 plates (Biolog, Inc., Hayward, CA, United States) as described previously (Essarioui et al., 2017). Briefly, spore suspensions (OD_590_ = 0.2) were used to inoculate individual Biolog SF-P2 plates following the manufacturer’s instructions. The inoculated plates were incubated for 72 h at 28°C. Growth and utilization of each of the 95 carbon compounds on the Biolog SF-P2 plate was estimated by measuring the absorbance of each well at 590 nm using a BioTek Microplate reader (Winooski, VT, United States). The measurement of absorbance is a direct measurement of increased turbidity and reflects actual microbial growth. The absorbance of the water control well was subtracted from the absorbance reading of each well to standardize absorbance values. Those nutrients with a resulting absorbance value greater than 0.005 were considered as “used nutrients” (Vaz Jauri et al., 2013). Niche width was defined as the total number of nutrients used by each isolate, and total growth as the sum of the absorbance values over all nutrients used. These values were used to calculate growth efficiency (total growth/niche width = mean growth per used nutrient) for each isolate.

To evaluate the potential for resource competition, niche overlap of each isolate against every other isolate in pairwise combination was determined for all isolates within each treatment using the formula:

$$\begin{array}{c} Niche\;overlap\;\left(Y\;against\;X\right)=\\ \sum_{i=1}^{95}95(min\left[X_{i},Y_{i}\right]/X_{i})\;/\;\left(niche\;width\left[isolate\;X\right]\right)\;\; \end{array}$$

Briefly, *X_i_* and *Y_i_* represent the growth (absorbance) of isolates *X* and *Y*, respectively, on carbon source *I*, and *(min [X_i_, Y_i_]/X_i_)* represents the proportional overlap of isolate *Y* against isolate *X* for nutrient *I*. The proportional overlap values are summarized across the 95 nutrients and scaled by the niche width (# of used nutrients) of isolate *X.* The resulting niche overlap value measures the average fraction of the growth of isolate *X* in competition with *Y* on nutrients utilized by both *X* and *Y*. Niche overlap values reflect the potential for nutrient competition between the two isolates.

### Antibiotic Inhibitory Interactions

Inhibitory interactions were evaluated for all possible pairwise isolate combinations among a randomly selected set of 10 isolates from each of the low dose carbon-amendment treatments (glucose, fructose, and complex) and the non-amended control (*n* = 40 isolates). In total, 1560 pairwise interactions were evaluated using an agar-overlay method (Kinkel et al., 2014). Briefly, 4 μl of spore suspension [∼10^8^ colony forming unit (CFU) per ml] of each “source” isolate was dotted onto an SCA plate (4 isolates/plate) and incubated at 28^°^C for 3 days. Dotted isolates were killed by inverting the uncovered Petri plates over 4 ml of chloroform in a watch glass for 1 h. Plates were then moved to a laminar flow hood and aerated for 30 min to allow the evaporation of residual chloroform. Each isolate pair was replicated twice on two different plates. Plates were subsequently overlaid with 10 ml of SCA and inoculated with 150 μl (∼10^8^ CFU/ml) of a “target” isolate spread uniformly over the surface of the agar plate using a L-shaped spreader, resulting in a dense lawn of the overlay isolate. Plates were incubated at 28°C for 3 days and then evaluated for zones of inhibition surrounding each source isolate. The size of any zone of growth inhibition was measured in millimeters by taking the average of two measurements, each taken from the edge of the dotted colony to the edge of the cleared zone with the two measurements taken being perpendicular to one another. Only inhibition zones ≥ 2 mm were considered to be inhibitory, representing the lack of growth of a target isolate when cultured in association with a source isolate. Interactions were categorized as resistant in the absence of an inhibition zone. The intensity of each inhibitory interaction was measured as the inhibition zone size (mm) of one target isolate by a source isolate.

### Data Analyses

All data analyses were conducted using R statistical software R version 3.2.0 (R Core Team, 2015). The vegan package for R (version 2.2-1) was used to conduct multidimensional analyses. A Euclidean dissimilarity matrix incorporating optical density values for each isolate on all 95 nutrients included in the Biolog SF-P2 plates was used to create non-metric multidimensional scaling (NMDS) plots. Ellipses, capturing percent of variation in nutrient use among isolates, were constructed using the function “ordiellipse,” and ADONIS was used to calculate treatment *r*^2^ and *p*-values. Analysis of variance (ANOVA), least significant difference test (LSD) and *T*-test, all with *p* < 0.05 as the significance level, were used to analyze differences between treatments in nutrient use, and inhibition and resistance phenotypes. Additionally, linear regression was used to characterize the relationships between nutrient use, and antibiotic inhibitory and resistant phenotypes. Proportions of inhibitory and resistant interactions were arcsine square root-transformed prior to analysis of variance and regression analysis.

## Results

### Nutrient Use Profiles of *Streptomyces*

**FIGURE 1:**
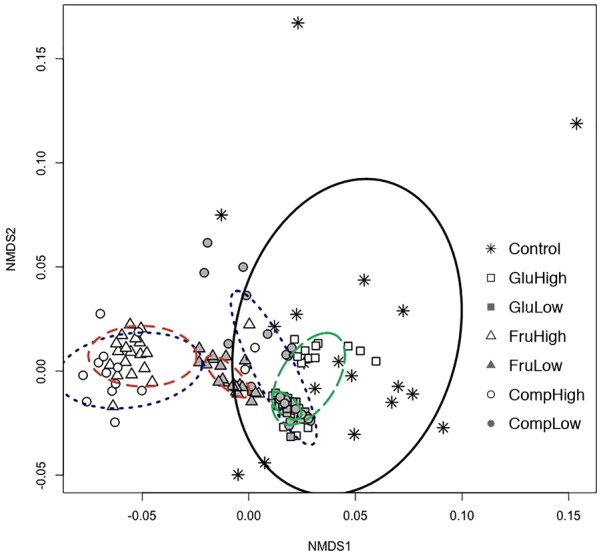
Non-metric multidimensional scaling (NMDS) based on carbon nutrient use profiles in Biolog SF-P2 plates for *Streptomyces* isolates from carbon-amended (glucose-high dose, open square; glucose-low dose, solid square; fructose-high dose, open triangle; fructose-low dose, solid triangle; complex-high dose, open circle; complex-low dose, solid circle) and non-amended (control, asterisk) soils after 72 h incubation period. Ellipses connect and define the centroids of communities from different carbon amendment treatments (glucose, green and long-dashed; fructose, orange, and dashed; complex, blue and dotted; non-amended control, black and solid).

Nutrient use profiles of *Streptomyces*, which reflect differences in the strength of preference for certain nutrients, varied among isolates from carbon-amended and non-amended soils (ADONIS: *r^2^* = 0.44, *p* < 0.005; Figure 1). Carbon amendments selectively enriched a subset of isolates with more similar nutrient use profiles from the total array present in non-amended soils. Specifically, isolates from the non-amended control soil were more variable in nutrient use, while isolates from amended soils tended to cluster together with other isolates from the same treatment. However, characterization of each isolate based on their ability to use or not each individual nutrient demonstrated that the vast majority of *Streptomyces* isolates (97%) across all treatments exhibited unique nutrient use phenotypes.

Both the type and dose of the carbon amendment influenced nutrient use among *Streptomyces*. For all nutrients, there was a clear separation between isolates from soils amended with the same carbon source at the high dose versus the low dose (Figure 1). This separation was particularly evident among isolates from soils treated with the complex amendment, and was less apparent among isolates from soils treated with glucose or fructose. Within the single nutrient treatments, the low dose amendment resulted in populations with more uniform nutrient use profiles than the high dose amendment. Interestingly, addition of a complex amendment reversed this pattern and more variability was observed among isolates from the low dose versus the high dose treatment. Comparing carbon sources, soil amendment with fructose resulted in the greatest difference in nutrient use profiles of *Streptomyces* isolates compared to the non-amended control. In contrast, isolates from the glucose-amended soils generally had nutrient use profiles most similar to isolates from the non-amended control.

### *Streptomyces* Niche Width and Growth Efficiency

**FIGURE 2:**
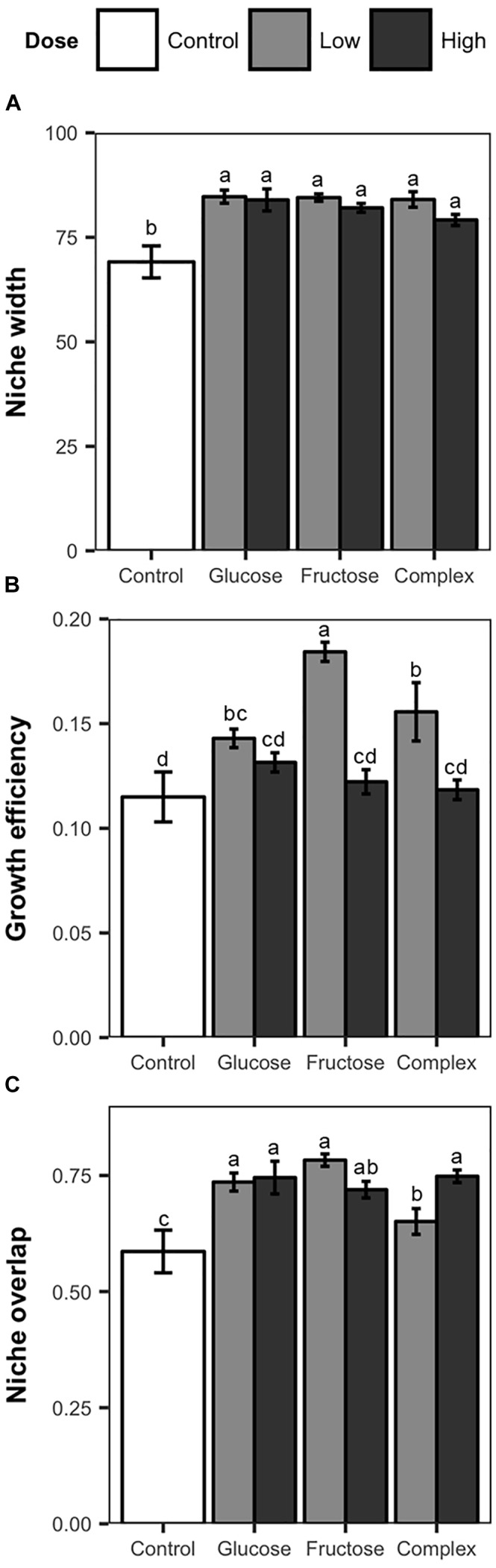
Nutrient use measurements, including mean niche width **(A)**, mean growth efficiency **(B)**, and niche overlap **(C)** of *Streptomyces* isolates from carbon-amended and non-amended soils after 72 h incubation period based on the utilization of 95 distinct carbon sources determined using Biolog SF-P2 plates. Mean niche width represent the number of substrates utilized. Mean growth efficiency was calculated by dividing total growth (sum of the absorbance values over all nutrients used) by niche width for each isolate. Mean niche overlap was determined for all possible pairwise *Streptomyces* isolate combinations from same treatment. Within each figure, different letters above the bars indicate significant differences among treatments. Bars sharing at least one letter are not significantly different from each other (ANOVA and LSD test, *p* < 0.05). Error bars represent standard errors.

Addition of carbon amendments significantly increased soil total carbon in all treatments (Table 1). Additionally, soil carbon amendments, despite dose or type altered niche width among soil *Streptomyces.* Overall, individual *Streptomyces* isolates used an average of 80 of the 95 substrates in the Biolog SF-P2 plates. However, average niche widths varied significantly among *Streptomyces* isolates from carbon-amended and non-amended treatments (83 vs. 69, respectively, ANOVA, *F* = 13.03, *p* < 0.05). Although mean niche widths were significantly greater among isolates from carbon-amended soils, no significant differences were observed in niche widths among different carbon amendments or doses (Figure 2A). Consistent with the pattern observed in the overall nutrient use profiles, there was much greater variation in niche width among isolates from the non-amended control than isolates from carbon-amended soils. Specifically, the number of nutrients used by isolates from non-amended soils ranged from 46 to 92, with half of the isolates using fewer than 60 of the substrates provided. On the other hand, the niche widths of isolates from carbon-amended treatments ranged from 68 to 93.

**Table 1:** Mean total carbon (TC) content in carbon-amended and non-amended soils.

Treatment		TC (%)	SE
Carbon amendment	Dose^∧^		
Complex^∗^	High	3.80^a^	0.49
Fructose	High	3.30^ab^	0.11
Glucose	High	2.66^b^	0.36
Complex	Low	1.90^c^	0.09
Fructose	Low	1.86^c^	0.12
Glucose	Low	1.77^c^	0.21
Control (non-amended)	–	1.25^e^	0.06


*Soil samples were collected from each mesocosm after 9 months incubation, and TC was measured by dry combustion. The TC values presented represent the mean of soil samples from at least four replicated mesocosms, and the corresponding standard errors (SE). Significant differences among treatments are indicated by different letters (ANOVA and LSD test, *p* < 0.05). ^∗^Complex carbon amendment treatment consisted of a combination of glucose, fructose, and malic acid (Acros Organics). ^∧^Dose: low dose amendments were equivalent to 250 g C/m^*2*^/year, and the high dose amendments equivalent to 750 g C/m^*2*^/year*.

Growth efficiency (total growth/niche width) of soil *Streptomyces* also varied in response to carbon amendments (Figure 2B). Significant differences in growth efficiency were observed between isolates from carbon-amended and non-amended soils (0.14 vs. 0.11, respectively, ANOVA, *F* = 4.71, *p* < 0.05). Overall, *Streptomyces* isolates from carbon-amended soil had growth efficiencies on average 5% higher than isolates from the non-amended control. *Streptomyces* isolates from low dose carbon-amended communities had greater growth efficiency than isolates from high-dose and non-amended communities (low = 0.16, high = 0.12 and non-amended = 0.11; ANOVA, *F* = 29.48, *p* < 0.05).

### *Streptomyces* Niche Overlap

Niche overlap among isolates within a treatment differed between carbon-amended and non-amended treatments (ANOVA, *F* = 6.08, *p* < 0.05). As anticipated based upon NMDS results (Figure 1), isolates from any of the carbon-amended treatments had significantly greater niche overlap than those from the non-amended control (0.73 vs. 0.59, respectively; Figure 2C). Among carbon-amended treatments, isolates from the single nutrient treatments had larger niche overlap with other isolates from the same treatment than did isolates from within complex amendment treatment (glucose = 0.74, fructose = 0.75, and complex = 0.69; ANOVA, *F* = 8.92, *p* < 0.05). In contrast, there were no significant differences in niche overlap among isolates from high versus low dose carbon amendment treatments, though, isolates from these treatments had larger niche overlap than those from the non-amended control (high = 0.74, low = 0.72, and control = 0.58; ANOVA, *F* = 10.51, *p <* 0.05).

### *Streptomyces* Inhibitory and Resistant Phenotypes

**FIGURE 3:**
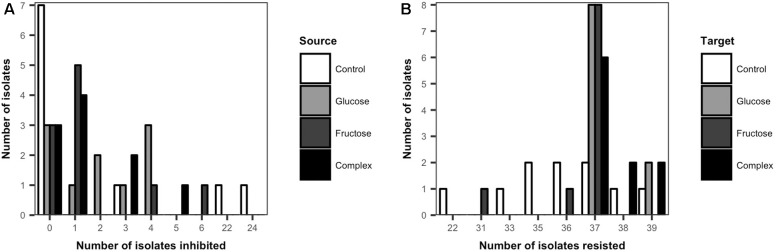
Frequency distribution of cumulative inhibitory **(A)** and resistant **(B)** phenotypes of *Streptomyces* from carbon-amended and non-amended soils. Each bar represents the number of source *Streptomyces* isolates (*n* = 10 isolates per treatment) that inhibited any of the *Streptomyces* target isolates from all the treatments (*n* = 39) **(A)**, or the number of target *Streptomyces* isolates (*n* = 10 isolates per treatment) that resisted inhibition by any of the source *Streptomyces* isolates from all the treatments (*n* = 39) **(B)**. Inhibitory interactions were determined by the presence of complete inhibition zones (>2 mm) representing the lack of growth of a target isolate in association with a source isolate on agar plates. Resistant interactions were determined by the presence and size of incomplete inhibition zones (<2 mm) representing the growth of a target isolate and the lack of inhibition by a source isolate on agar plates.

The inhibitory and resistance capacities of *Streptomyces* populations were evaluated among all isolates from low dose carbon-amended and the non-amended treatments. Overall, the majority of *Streptomyces* isolates (60%) inhibited at least one other *Streptomyces* isolate (Figure 3A). Among the 60% of isolates that could inhibit at least one other isolate, the average number of isolates inhibited varied significantly between carbon-amended and non-amended treatments (non-amended = 16.3, glucose = 2.9, fructose = 2.1, complex = 2.1, ANOVA, *F* = 8.23, *p* < 0.05). While the average number of isolates inhibited was lower in the carbon-amended vs. non-amended treatment, the largest number of non-inhibitory *Streptomyces* isolates was also observed in the non-amended control (7 out of 10). However, two of the three inhibitory isolates from the non-amended soil were the most inhibitory of all isolates collectively, inhibiting 22 and 24 of the 39 other isolates (Figure 3A).

No major differences were observed in the inhibitory activity among *Streptomyces* isolates from the different carbon-amended treatments. In contrast to non-amended soils, most (7 out of 10) of the *Streptomyces* isolates from each of the carbon-amended treatments inhibited at least one *Streptomyces* isolate. However, these isolates inhibited fewer *Streptomyces* isolates (range: 1–6) than the two highly inhibitory phenotypes from the non-amended control (Figure 3A).

All *Streptomyces* isolates resisted inhibition by a large number of other *Streptomyces* isolates (Figure 3B). There were significant differences in *Streptomyces* resistance phenotypes among isolates from carbon-amended and non-amended treatments (ANOVA, *F* = 7.47, *p* < 0.05). The greatest variability among isolates in resistance capacity was observed in the non-amended control. Here, frequencies of resistance were approximately normally distributed (range from 22 to 39 resistant isolates), with isolates resistant to an average of 35 isolates. On the other hand, resistance phenotypes among *Streptomyces* isolates from carbon-amended treatments were less variable, and 95% of the isolates resisted 37–39 isolates.

### Frequency of *Streptomyces* Inhibitory and Resistant Interactions Among Treatments

**FIGURE 4:**
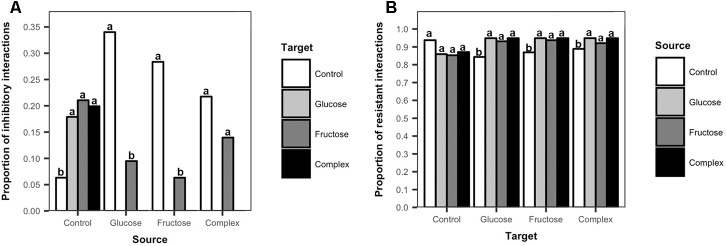
Frequency of inhibitory and resistant interactions among *Streptomyces* isolates from carbon-amended and non-amended soils. Each bar represents the mean proportion of inhibitory interactions among source *Streptomyces* isolates (*n* = 10) that were inhibitory against target *Streptomyces* isolates (*n* = 10) from different treatments **(A)**, or the mean proportion of resistant interactions among target *Streptomyces* isolates (*n* = 10) that resisted inhibition by source *Streptomyces* isolates (*n* = 10) **(B)**. Inhibitory interactions were determined by the presence and size of complete inhibition zones (>2 mm) representing the lack of growth of a target isolate in association with a source isolate on agar plates. Resistant interactions were determined by the presence and size of incomplete inhibition zones (<2 mm) representing the growth of a target isolate and the lack of inhibition by a source isolate on agar plates. Different letters above the bars indicate significant differences among treatments of source isolates (Figure 4A) and within treatments of target isolates (Figure 4B). Bars sharing at least one letter are not significantly different from each other (ANOVA and LSD test, *p* < 0.05).

To further explore the effects of carbon additions to soil on *Streptomyces* inhibitory and resistant phenotypes, the frequencies of inhibition and resistance among isolates from carbon-amended and non-amended treatments were evaluated. There was a clear separation in the inhibitory profiles of isolates from carbon amended versus non-amended soils (Figure 4A). *Streptomyces* isolates from non-amended soils inhibited a larger proportion of isolates from any carbon-amended soils than did isolates from the same treatment (ANOVA, *F* = 0.6617, *p* < 0.05, Figure 4A). Only isolates from the non-amended control were inhibitory against *Streptomyces* isolates from the glucose or complex amendment treatments. In contrast, *Streptomyces* from carbon-amended soils were predominantly inhibitory against isolates from non-amended soils, and against a smaller number of isolates from fructose-amended soils, though inhibitory isolates from carbon-amended soils inhibited more *Streptomyces* from non-amended than from fructose-amended soils (glucose, ANOVA, *F* = 12.33, *p* < 0.05; fructose, ANOVA, *F* = 9.95, *p* < 0.05; complex, ANOVA, *F* = 5.04, *p* < 0.05, Figure 4A). These results suggest that carbon amendments have considerable impacts on soil *Streptomyces*, selecting for populations that were less sensitive to antibiotic inhibition.

Differences in resistant interactions were also observed in response to soil carbon amendments (Figure 4B). *Streptomyces* isolates from carbon-amended soils were significantly more resistant to inhibition by isolates from any of the carbon-amended soils, than to isolates from non-amended soils (glucose, ANOVA, *F* = 36, *p* < 0.05; fructose, ANOVA, *F* = 6.48, *p* < 0.05; complex, ANOVA, *F* = 27.15, *p* < 0.05, Figure 4B). In contrast, among isolates from the non-amended control, there were no significant differences in their resistance to isolates from non-amended or carbon-amended soils (ANOVA, *F* = 1.28, *p* > 0.05, Figure 4B). These results indicate that there is a consistent amplification of resistance among *Streptomyces* populations in response to soil carbon amendments, primarily selecting for phenotypes that resist inhibition by other *Streptomyces* from carbon-amended soils that may have similar functional characteristics.

### Relationships Between Nutrient Use, Inhibition, and Resistance Among *Streptomyces*

**FIGURE 5:**
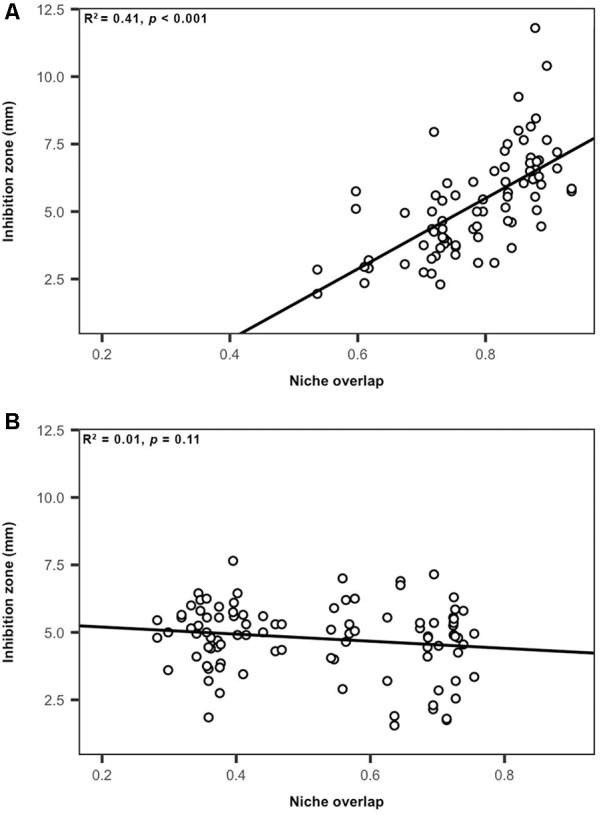
Relationship between nutrient use and intensity of inhibition among *Streptomyces* isolates from carbon-amended and non-amended soils. **(A)** *Streptomyces* isolates from carbon-amended soils that were inhibitory against isolates from non-amended soils, **(B)** *Streptomyces* isolates from non-amended soils that were inhibitory against isolates from carbon-amended soils. Each circle represents the relationship between niche overlap and inhibition zone size (mm) corresponding to individual source *Streptomyces* isolates that were inhibitory to target *Streptomyces* isolates **(A)** 81, inhibitory interactions, **(B)** 97, inhibitory interactions.

To evaluate how carbon amendments influence competitive interactions among *Streptomyces*, we considered the relationships between niche overlap (nutrient-use) and inhibition intensity for all inhibitory pairs of *Streptomyces* isolates. There was a significant (but small) positive correlation between niche overlap and inhibition intensity when considering inhibitory interactions between *Streptomyces* pairs from all treatments (*R^2^* = 0.02, *F* = 6.6, *p* < 0.05, Supplementary Figure S1). However, the strength of this relationship varied when separating interactions between *Streptomyces* isolates from carbon-amended soils that were inhibitory against isolates from non-amended soils and those interactions between *Streptomyces* isolates from non-amended soils that were inhibitory against those isolates from carbon-amended treatments (Figure 5). For example, niche overlap explained 41% of the total variation in inhibition zone sizes among *Streptomyces* from carbon-amended soils against isolates from the non-amended control (*R^2^* = 0.41, *F* = 57.4, *p* < 0.05, Figure 5A). In contrast, there was no significant relationship between niche overlap and inhibition zone size for *Streptomyces* from non-amended soils against isolates from carbon-amended treatments (*R^2^* = 0.01, *F* = 2.5, *p* > 0.05, Figure 5B). These results suggest that in carbon-amended soils, there has been selection for *Streptomyces* populations that inhibit more intensively other *Streptomyces* that may represent nutrient competitors.

## Discussion

In this study, we hypothesized that the addition of carbon compounds to soil would impose selection that would result in shifts in nutrient use profiles, and antibiotic inhibitory and resistance phenotypes, of *Streptomyces* populations in agricultural soils.

Carbon inputs to soil selectively enriched *Streptomyces* populations having more similar nutrient use profiles than their counterparts from non-amended soils. Low dose inputs of a single carbon source selected for more uniform populations in comparison to communities from soils treated with high dose or complex amendments. Other studies have also reported significant differences in resource utilization patterns between microbial communities from soils amended with simple compounds versus complex carbon substrates such as plant residues and more recalcitrant materials (Degens and Harris, 1997; Schutter and Dick, 2001). Our results provide further evidence of the effect of soil carbon amendments in selecting for populations with substantial similarity in their substrate utilization profiles, and constraining variation in metabolic diversity of soil *Streptomyces*. Thus, the selective effect of soil carbon amendments may plausibly intensify nutrient competition among co-existing populations.

Addition of carbon amendments resulted in significant increases in soil total carbon, as well as increased niche widths and growth efficiencies among indigenous soil *Streptomyces*. The increases in niche width and growth efficiency may reflect shifts in soil carbon dynamics and pools, and selection for *Streptomyces* populations that utilize a broader range of resources that may become more abundant after the addition of soil amendments. These results are consistent with previous studies reporting differences in niche width and growth of *Streptomyces* populations from soils varying in organic carbon pools (Schlatter and Kinkel, 2014; Essarioui et al., 2016). Addition of labile carbon compounds to soil mesocosms may also have increased overall microbial community activity, and the release of microbial-derived compounds (Kallenbach et al., 2016), which may have altered the composition and availability of resources in amended soils. Interestingly, although soil total carbon increased with dose of carbon amendments, significant differences in *Streptomyces* growth efficiency in comparison to the non-amended control were observed only in response to the addition of low dose amendments. These findings are consistent with results observed in previous studies (Bremer and Kuikman, 1994; Schlatter et al., 2009; Bastida et al., 2013).

In general, theory predicts that high resource abundance favors niche-generalists over niche-specialists adapted to compete and survive when resources are limited (Pianka, 1970). In this study, both high and low dose carbon amendments largely selected for generalist phenotypes that were able to use a wider range of carbon sources, while the more niche-specialist phenotypes (narrow niche width) were predominant in non-amended soils. Similar results were observed in previous studies in which the relative abundance of major soil bacterial phyla characterized as soil generalists increased in response to sucrose amendments, and these bacterial groups were more abundant in rhizosphere soils than in bulk soils with lower carbon availability (Fierer et al., 2007). Moreover, our data are in line with findings from recent work showing differences in temporal growth dynamics and biomass accumulation among *Streptomyces* from polycultures and their counterparts from low-nutrient monocultures (Essarioui et al., 2016). Collectively, these data offer insight into how soil carbon amendments can lead to significant shifts in microbial nutrient use and growth strategies to optimize fitness and adaptation to local resource dynamics in the soil. It is therefore possible that *Streptomyces* populations from carbon-amended soils may reflect those populations that are predominant in high-nutrient environments such as the rhizosphere, while the populations from non-amended soils may be more similar to those in nutrient-limited conditions such as bulk soils.

While greater niche overlap was observed among isolates from carbon-amended soils, greater niche differentiation was observed among isolates from non-amended soils. Niche overlap in carbon-amended soils partially reflects the result of niche broadening, and suggests an intensification of nutrient competition among potential competitors (Bulleri et al., 2016; Essarioui et al., 2016). Soil carbon amendments to soil resulted in *Streptomyces* populations with more complex metabolic capacities, and although they can use a wider range of nutrients, they may preferentially use a subset of nutrients for which their availability could vary in response to carbon additions to soil. At the same time, the coincidence of greater niche differentiation and variability in nutrient use profiles with narrower niche width, suggests that depletion of nutrients in non-amended soils may have selected for *Streptomyces* phenotypes that are specialized on distinct subsets of nutrients as a means of minimizing resource competition.

Shifts in microbial nutrient use and growth strategies may coincide with positive selection for *Streptomyces* antibiotic inhibitory phenotypes in nutrient-amended soils (Schlatter et al., 2009; Wiggins and Kinkel, 2005a,b). In our study, carbon amendments resulted in *Streptomyces* populations with phenotypes that combine both antagonistic capacities and a generalist lifestyle. Antagonistic traits may be critical to allowing niche*-*generalists to inhibit more efficient niche-specialists in the soil (Russel et al., 2017). In fact, antagonist *Streptomyces* from carbon-amended soils were predominantly inhibitory against isolates from non-amended soils, which had notably smaller niche widths. The positive relationship between niche overlap and inhibition intensity between inhibitory and susceptible *Streptomyces* from carbon-amended and non-amended soils, respectively, suggests that inhibitory phenotypes among generalists were selectively enriched against the strongest resource competitors.

*Streptomyces* from non-amended soils had smaller niche widths regardless their ability to inhibit other isolates. However, *Streptomyces* with high, versus none, inhibitory capacities did not differ significantly in niche width or growth efficiency, suggesting that reductions in nutrient use and growth may just reflect the selection for *Streptomyces* phenotypes with niche-specialist attributes, and not fitness tradeoff associated with antibiotic inhibition (Schlatter and Kinkel, 2015). The higher frequency of non-inhibitory phenotypes in non-amended soils also hints at the possibility that the cost of maintaining inhibitory traits may become substantial in carbon-limited environments, and could lead to an accelerated reduction or loss of these traits. The ability to predominantly inhibit *Streptomyces* isolates from carbon-amended soils suggests that rather than producing multiple distinct antibiotics, the broad antagonistic property of the two highly inhibitory *Streptomyces* from non-amended soils may be the result of producing a novel antibiotic for which the metabolically similar *Streptomyces* from carbon-amended soils lacked immunity. However, the selective forces or adaptation traits that allowed these inhibitory phenotypes to be maintained in non-amended soils remain unknown.

In previous studies, the resistance to antibiotic inhibition was common among *Streptomyces* from both carbon-amended and non-amended soils (Davelos et al., 2004; Kinkel et al., 2014; Schlatter and Kinkel, 2015). In the non-amended control in our study, there were few extreme phenotypes (weak and highly resistant), and most *Streptomyces* had an intermediate resistance capacity. In contrast, the vast majority of *Streptomyces* isolates from the carbon-amended soils were resistant to virtually all the other *Streptomyces*.

Highly resistant *Streptomyces* also had broader niche widths, suggesting that resistance did not impose strong tradeoffs in resource use. These results contradict those of a previous study in which highly resistant *Streptomyces* from natural prairie soils had less efficient growth and utilized a smaller number of resources for growth than those with low resistance capacities (Schlatter and Kinkel, 2015). Our results also suggest that shifts in antibiotic sensitivity and production induced by the incorporation of carbon amendments to the soil, could result in the emergence of ecological distinct populations with distinct life history strategies, functional characteristics, species interactions, and, consequently, co-evolutionary dynamics (Nesme and Simonet, 2015).

Nutrient competition has been described as one of the main drivers of antagonistic interactions among metabolically similar species (Case and Gilpin, 1974). Therefore, greater antibiotic inhibition was expected among *Streptomyces* populations from carbon-amended soils that had greater niche overlap and stronger similarity of nutrient use profiles. However, instead of greater antibiotic inhibition, we observed an accumulation of resistance to metabolically similar populations in response to carbon additions to soil. While highly resistant to similar phenotypes, *Streptomyces* from carbon-amended soils showed sensitivity to inhibition by *Streptomyces* from non-amended soils, which were characterized by more diverse nutrient use, inhibitory, and resistance phenotypes. The substantial differences in functional traits observed between *Streptomyces* from carbon-amended and non-amended soils, suggest that although these populations originated from the same soil, the action of receiving or not a carbon amendment over the course of 9 months acted as a selective force that led to significant phenotypic shifts. These shifts in *Streptomyces* metabolic, inhibitory and resistance repertoires may reflect the adaptation of species interaction phenotypes to distinctive selection pressures imposed by the recurrent addition of carbon resources to the soil, and the subsequent changes in soil resource dynamics and pools.

In closing, our findings demonstrate that soil carbon amendments impose strong selection on indigenous soil *Streptomyces*. These results shed light on the potential for using carbon amendments to strategically mediate nutrient competition and antagonistic interactions, and foster the assembly of thriving soil microbial communities performing functions of importance to agriculture. However, further studies are necessary to elucidate the impact of changes in *Streptomyces* nutrient use and antibiotic inhibitory phenotypes on plant pathogen suppression in agricultural soils.

## Author Contributions

JPD-A performed the experiments, analyzed and interpreted the data, and wrote the manuscript. JPD-A and LF performed the Biolog analyses. LF contributed to the design of the experiments, established the soil mesocosms and isolated *Streptomyces* isolates. RD-M contributed to the conception of the work and design of the experiments. LK contributed to the conception of the work and design of the experiments, oversaw the project and helped with data analysis and manuscript preparation.

## Conflict of Interest Statement

The authors declare that the research was conducted in the absence of any commercial or financial relationships that could be construed as a potential conflict of interest.

## Supplementary Material

FIGURE S1 Relationship between niche overlap and inhibition zone size (mm) among *Streptomyces* isolates from carbon-amended and non-amended soils. Each circle represents the relationship between the niche overlap and inhibition zone size (mm) corresponding to individual source *Streptomyces* isolates that were inhibitory to target *Streptomyces* isolates from any treatment (*n* = 178 inhibitory interactions).
