# Supplementary figures and images for: Carbon Amendments Induce Shifts in Nutrient Use, Inhibitory, and Resistance Phenotypes Among Soilborne Streptomyces
Source: Front Microbiol. 2019 Mar 27;10:498. doi: 10.3389/fmicb.2019.00498 (PMC6445949; doi:10.3389/fmicb.2019.00498)

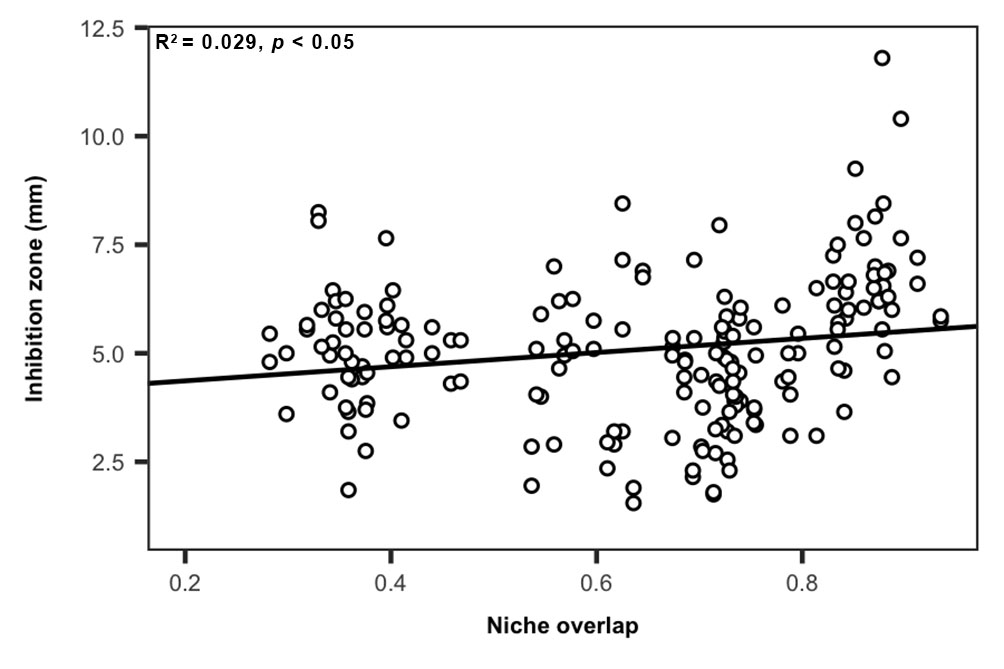

Supplement: FIGURE S1 — Relationship between niche overlap and inhibition zone size (mm) among Streptomyces isolates from carbon-amended and non-amended soils. Each circle represents the relationship between the niche overlap and inhibition zone size (mm) corresponding to individual source Streptomyces isolates that were inhibitory to target Streptomyces isolates from any treatment (n = 178 inhibitory interactions). [file Image_1.JPEG]
